# Clinical Characteristics of Paracentral Acute Middle Maculopathy in Eyes with Retinal Vascular Occlusion Diseases in Chinese Patients

**DOI:** 10.1155/2021/8867570

**Published:** 2021-06-18

**Authors:** Zhengwei Zhang, Yunjia Jiang, Xiaoli Huang, Zhifeng Wu, Bilian Ke

**Affiliations:** ^1^Department of Ophthalmology, Shanghai General Hospital of Nanjing Medical University, Shanghai, China; ^2^Department of Ophthalmology, The Affiliated Wuxi No. 2 People's Hospital of Nanjing Medical University, Wuxi, Jiangsu Province, China; ^3^Department of Ophthalmology, Wuxi No. 2 People's Hospital, Affiliated Wuxi Clinical College of Nantong University, Wuxi, Jiangsu Province, China; ^4^Department of Ophthalmology, Shanghai General Hospital, School of Medicine, Shanghai Jiao Tong University, Shanghai, China; ^5^National Clinical Research Center for Eye Diseases, Shanghai, China; ^6^Shanghai Key Laboratory of Fundus Disease, Shanghai, China; ^7^Shanghai Engineering Center for Visual Science and Photomedicine, Shanghai, China; ^8^Shanghai Engineering Center for Precise Diagnosis and Treatment of Eye Diseases, Shanghai, China

## Abstract

**Aim:**

To investigate the incidence and clinical characteristics of paracentral acute middle maculopathy (PAMM) and its relationship with prominent middle limiting membrane (p-MLM) sign in eyes with retinal artery occlusion (RAO) or retinal vein occlusion (RVO) in a Chinese clinical setting.

**Methods:**

In this retrospective observational study from January 2015 to May 2020, multimodal imaging data of 807 eyes including 555 consecutive patients with RVO or 252 consecutive patients with RAO were reviewed. All patients were scanned using the spectrum-domain optical coherence tomography (OCT), and some of them underwent color fundus photography, fundus fluorescence angiography, en face OCT, and OCT angiography.

**Results:**

PAMM was detected in 49 eyes of 49 RAO patients and 29 eyes of 29 RVO patients. The mean ages at presentation were 64.49 ± 13.90 years and 54.00 ± 18.48 years in RAO and RVO patients (*P*=0.006), respectively. Eyes with RAO were more prone to develop PAMM (19.44% [49/252] vs. 5.23% [29/555]; *P* < 0.001). Of the 78 eyes with PAMM, 24 eyes (7 eyes with RVO and 17 eyes with RAO) were found with p-MLM sign. An interesting phenomenon that had been overlooked before was that the hyperreflective line of the p-MLM sign was usually continuous, regardless of the type of PAMM lesion.

**Conclusions:**

This series is the largest to date to describe the clinical characteristics of PAMM and p-MLM sign in Chinese patients. The incidence of PAMM and p-MLM sign in patients with RAO was relatively higher than that in patients with RVO. These signs alone probably represent milder ischemia and prompt us to carry out a comprehensive and meticulous examination to prevent the further development of the disease. In addition, the hyperreflective line of the p-MLM sign was usually continuous, which could support the totally venous nature of the retinal deep capillary plexus to some extent.

## 1. Background

Paracentral acute middle maculopathy (PAMM), which was first defined by Sarraf et al. in 2013, is characterized by the presence of a hyperreflective band spanning the inner nuclear layer (INL) with subsequent permanent INL thinning [1]. It is being increasingly reported and demonstrated not to be a distinct entity but a common sign of several ocular diseases [2], uneventful ocular surgeries [3, 4], or even systemic diseases [5–7]. Although its pathophysiology is not yet fully understood, impaired perfusion through the retinal capillary system, leading to hypoperfusion or ischemia of the deep vascular complex (DVC), has been proven to play a major role [8]. The DVC comprises two deeper capillary networks, located above and below the INL, that are supplied by vertical anastomoses from the superficial vascular plexus [9]. It is well acknowledged that the mechanism associated with PAMM is primarily due to sublethal ischemic hypoxia of the middle retinal tissue, mainly the layer of INL [2, 10].

Also, in 2013, Chu et al. [11, 12] introduced the “prominent middle limiting membrane (p-MLM) sign,” a hyperreflective line in the inner synaptic portion of the outer plexiform layer (OPL) on spectral-domain optical coherence tomography (SD-OCT) B-scan images, as an indicator of acute ischemic retinal damage in retinal artery occlusion (RAO) and diagnostic tool to distinguish ischemic from the nonischemic retinal vein occlusion (RVO). The pathogenesis of the p-MLM sign is similar to that of PAMM; however, the relationship between them is poorly investigated.

PAMM is associated with RVO [13–16] and RAO [17, 18], including cilioretinal arterial occlusion [19, 20]. In the first and largest study of PAMM involving eyes with nonischemic central RVO (CRVO), approximately 5.17% (25/484) of the American patients with nonischemic CRVO showed PAMM on SD-OCT [13]. However, no large-scale study has reported the prevalence or clinical characteristics of PAMM in Chinese patients with RVO or RAO, although a few case reports and small case series exist [21–23]. Therefore, the purpose of this study was to investigate the incidence and clinical characteristics of PAMM and its relationship with the p-MLM sign in eyes with RVO or RAO in a Chinese clinical setting with the largest sample size to date. In addition, we aimed to infer the nature of the retinal deep capillary plexus through optical coherence tomography (OCT) features of PAMM and the p-MLM sign.

## 2. Methods

In this retrospective study, consecutive cases with a clinical diagnosis of any type of RAO or RVO (branch or central and ischemic or nonischemic) from January 2015 to May 2020 were included. The clinical diagnosis was based on retinal findings, patient symptoms, and supplemental evidence from fundus fluorescence angiography (FFA) findings. If an eye showed RVO and cilioretinal artery occlusion simultaneously, it was classified as having RVO for the cause analysis. Exclusion criteria were as follows: (1) medium too cloudy to be scanned with SD-OCT; (2) quality of the OCT image too poor to be evaluated; and (3) a history of eye surgery within one month before the first visit. The Institutional Review Board of the Affiliated Wuxi No.2 People's Hospital of Nanjing Medical University approved the protocol. This study complied with the tenets of the Declaration of Helsinki.

Each patient underwent complete ocular examination, including best-corrected visual acuity (BCVA) assessment, noncontact tonometry, slit-lamp examination, and indirect ophthalmoscopy. Retinal imaging was performed using a high-speed 840 nm-wavelength SD-OCT instrument RTVue XR Avanti (Optovue, Inc., Fremont, California, USA) or Cirrus HD-OCT with eye-tracking ability (Model 4000; Carl Zeiss Meditec, Inc., Dublin, CA). To detect the presence/absence of PAMM lesions and p-MLM sign, each OCT B-scan image was assessed by two researchers (Z.W.Z. and X.L.H.) in a blinded manner. Particularly, band-like hyperreflective lesions of the INL corresponded to PAMM lesions [1], and a hyperreflective line in the inner synaptic portion of the OPL corresponded to the p-MLM sign [11]. Disagreements regarding PAMM lesions and p-MLM sign were resolved by a third senior observer (Z.F.W.).

In some cases, en face OCT and OCT angiography were performed using the RTVue XR Avanti SD-OCT with AngioVue software (Optovue, Inc., Fremont, California, USA), which has a light source of 840 nm wavelength and 45 nm bandwidth with an A-scan rate of 70 kHz. A 3 × 3 mm scan or, 6 × 6 mm or cube scan centered on the fovea was acquired by two repeated B-scans at 304 raster positions, with each B-scan comprising 304 A-scans. Two volumetric raster scans with orthogonal fast-scan directions (horizontal and vertical) were acquired for each eye and merged to remove motion artifacts [24]. This has been described in detail in our previous work [25]. PAMM can be divided into three patterns of these areas on en face OCT [26]: arteriolar (band-like areas in the distribution of major arterioles), globular (ovoid focal or multifocal areas), and fern-like patterns (multilobulated central area tracking along veins).

In a few patients, color fundus photographs were acquired with a Topcon TRC-50IX color fundus camera (Topcon Medical Systems, Tokyo, Japan), and FFA was performed with Spectralis HRA (Heidelberg Engineering, Heidelberg, Germany).

Statistical analyses were performed using SPSS software version 21.0 (SPSS, Chicago, IL, USA). A one-sample Kolmogorov-Smirnov test was performed to assess the normality of distribution of continuous variables. Subsequently, a significance test was performed. When data were normally distributed, an independent-samples *t*-test was performed for group comparisons. Categorical data were compared using a Fisher's exact test. All tests were two-tailed, and *P* values <0.05 were considered to be statistically significant.

## 3. Results

Table 1 shows baseline patient demographics and ocular examination findings of patients with PAMM. Multiple imaging scans were reviewed for a total of 807 consecutive eyes of 807 patients, including 252 eyes with RAO (143 central RAO, 105 branch RAO, and four cilioretinal artery occlusion only) and 555 eyes with RVO (226 CRVO and 329 branch RVO). Seventy-eight eyes of 78 patients, with a mean age at onset of 60.59 ± 16.45 years (range: 20–84 years), demonstrated hyperreflective plaque-like lesions at the INL on cross-sectional SD-OCT B-scans, and these were regarded as PAMM lesions. Visual acuity ranged from 0 to 2.0 logMAR at the first clinical examination. Regarding preexisting systemic comorbidities, 41 patients had known histories of systemic arterial hypertension, six had open-angle glaucoma, 10 had diabetes mellitus, and the remaining 21 patients denied a history of systemic diseases.

Of these 78 patients with PAMM lesions, 49 eyes of 49 patients (49/252 or 19.44%; mean age: 64.49 ± 13.90 years; age range: 23–84 years) had RAO, and 29 eyes of 29 patients (29/555 or 5.23%; mean age: 54.00 ± 18.48 years; age range: 20–78 years) had RVO. Regarding the type of retinal vascular occlusion, there were 32 eyes with central RAO (32/143 or 22.38%), 17 eyes with branch RAO (17/109 or 16.0%; including two eyes with cilioretinal artery occlusion only), 22 eyes with CRVO (22/226 or 9.73%), and seven eyes with branch RVO (7/329 or 2.13%). Compared with patients with RVO, those with RAO were more prone to develop PAMM (19.44% vs. 5.23%; *P* < 0.001, Fisher's exact test) and older (64.49 ± 13.90 years vs. 54.00 ± 18.48 years; *P*=0.006; independent-samples *t*-test). The development of PAMM showed a male predilection (55 men, 70.5%; 23 women, 29.5%), but no left or right eye predilection (OD: 44 eyes; OS: 34 eyes).

**Figure 1:**
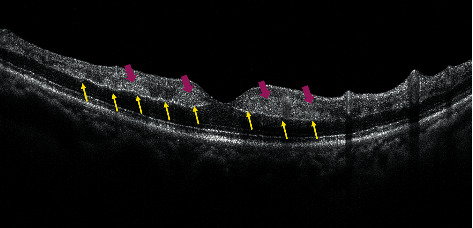
OCT displayed combined PAMM and p-MLM in one case with RVO. Pink arrows indicate skip PAMM lesions around the central macula. Yellow arrows indicate a continuous hyperreflective line being referred to as p-MLM sign on both sides of the fovea.

Of 78 eyes with PAMM, 24 (seven eyes with RVO and 17 eyes with RAO) also showed the p-MLM sign (24/78; 30.77%). All seven eyes with the p-MLM sign associated with RVO revealed skip PAMM lesions (Figure 1). However, in the RAO group, the p-MLM sign was associated with various types of PAMM lesions. Compared with patients without the p-MLM sign, patients with the p-MLM sign were significantly older (66.17 ± 11.26 vs. 58.11 ± 17.82 years; *P*=0.019; Table 2). However, no significant differences were found in terms of BCVA, sex, or eye distribution between the two groups (Table 2).

**Figure 2:**
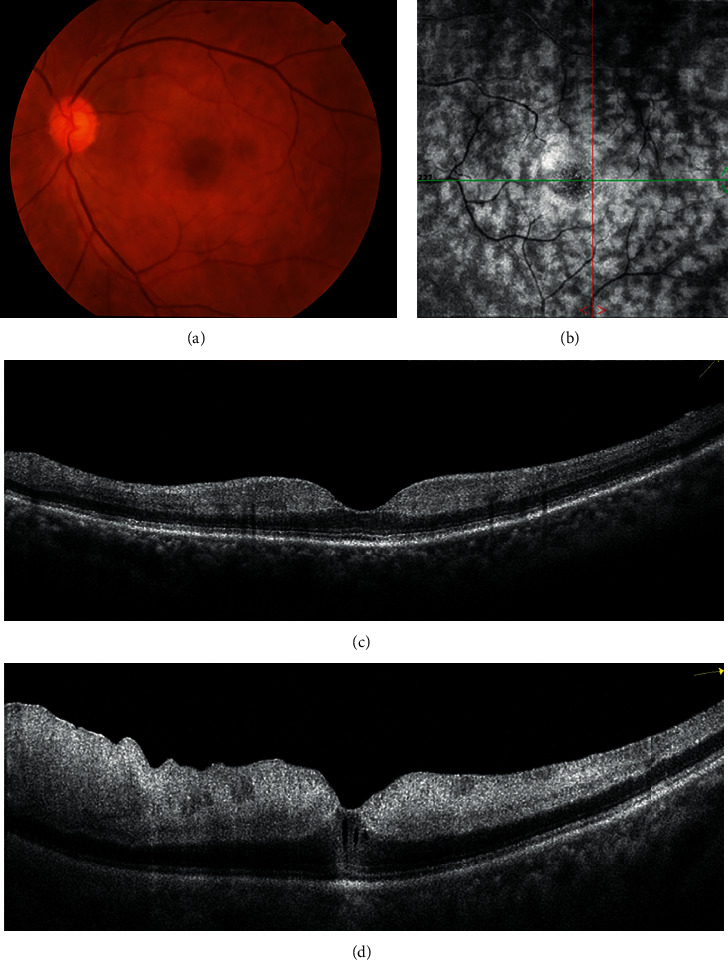
Multimodal imaging from a 55-year-old hypertensive man with partial CRAO and fern-like pattern PAMM at baseline and complete CRAO with diffuse middle and inner retinal ischemia. (a) Fundus photography illustrated perifoveal retinal whitening in the central macular region. (b) En face OCT illustrated a perivenular fern-like pattern with periarterial sparing. (c) PAMM lesions (hyperreflective bands at the level of INL) in a skip pattern revealed on the cross-sectional OCT B-scan. (d) One week later, the patient's vision declined to 2/200, and SD-OCT illustrated diffuse middle and inner retinal ischemia.

En face OCT images were available for 44 of 78 eyes (44/78 or 56.41%). En face OCT segmentation of the INL illustrated well-defined areas of hyperreflectivity corresponding to focal or multifocal PAMM on OCT B-scans. Three distinct patterns of PAMM lesions on en face OCT were observed: arteriolar in 24 eyes (24/44 or 54.54%), globular in seven eyes (7/44 or 15.91%), and fern-like in 13 eyes (13/44 or 29.55%).  Table 3 shows the detailed distribution of the three PAMM lesion patterns by etiology. Of 13 eyes with perivenular fern-like PAMM lesions, nine had RVO, whereas only four had RAO. One eye of central RAO with follow-up data displayed fern-like PAMM lesions with skip lesions in the INL at baseline, and subsequent progression of ischemia, diffusely involving the entire middle and inner retinal layers, was observed at the one-week follow-up visit (Figure 2).

## 4. Discussion

This was the largest study to date to characterize PAMM and first to show its association with the p-MLM sign in a Chinese clinical setting. As for the incidence of PAMM, we found that it was the most common in eyes with central RAO (32/143 or 22.38%) and the least common in eyes with branch RVO (7/329 or 2.13%). In eyes with RVO, the incidence of PAMM in this study (5.23%; 29/555) was consistent with that of the retrospective study conducted by Rahimy et al. (5.17%; 25/484) [13], although they only enrolled eyes with nonischemic CRVO. We were the first to assess the incidence of PAMM in eyes with RAO, which was as high as 19.44% (49/252). Consistently, in a retrospective, nonconsecutive, observational study, Yu et al. [27] identified PAMM in 22.5% (9/40) of cases with branch or central RAO, which was very close to our results. However, the incidence of PAMM in all cases of RVO and RAO may be much higher than that reported in this study because of the evanescent nature of PAMM lesions, which are covered by hemorrhage, or hyperreflectivity induced by severe ischemia of the whole inner retina. Nonetheless, our results could provide a general understanding of the clinical incidence of PAMM. More recently, Pichi et al. [20] reported that PAMM was present in 100% of the cases of isolated cilioretinal artery obstruction with or without CRVO, which may be because of hypoperfusion of the involved cilioretinal artery (as the author termed “cilioretinal artery insufficiency”), rather than complete occlusion. Together with the previous results, it is noteworthy that artery hypoperfusion or insufficiency resulted in more PAMM lesions compared to RVO.

Using en face OCT, three patterns of PAMM lesions were found, as described previously [26]. In our study, the most common pattern was arteriolar (24/44 or 54.54%), followed by fern-like (13/44 or 29.55%) and globular (7/44 or 15.91%). Therefore, although the fern-like pattern is distinctive, it is relatively common. The fern-like pattern of PAMM lesions correlates more strongly with RVO than RAO [15, 26]. In our study, 13 patients showed the perivenular fern-like PAMM lesion pattern, nine of whom had RVO and the remaining four had RAO (Table 3). Our results are consistent with previous observations that arterial hypoperfusion secondary to outflow obstruction from an RVO obstruction appears to be the most common cause of this presentation [15], and our study verified this phenomenon in a relatively large sample size. However, not all patients in the present study had undergone en face OCT, which may lead to omission of some cases of PAMM with the perivenular fern-like lesion pattern. Nonetheless, this possibility was minimized by identifying the skip pattern of PAMM lesions at the INL on cross-sectional SD-OCT, which is characteristic of perivenular fern-like PAMM lesion pattern [15, 28].

Since the concept of PAMM was widely accepted and applied in published articles, the usage of the p-MLM sign has been decreasing. The p-MLM sign could be present or absent and fails to account for the etiologic mechanism of deep retinal capillary ischemia; thus, it does not provide information about the extent of the ischemic damage [10, 27]. However, the pathogenesis of the p-MLM sign and its relationship with PAMM are still poorly understood. On OCT B-scans, the p-MLM sign was defined as a hyperreflective line, which had higher intensity of reflectivity compared with the adjacent normal retinal structures, located in the inner synaptic portion of the OPL. This probably develops due to cytoplasmic swelling of these synaptic portions of the OPL [11]. Similar to PAMM, the p-MLM sign could be found in cases of both RAO [11] and RVO [12, 29]. Moreover, both PAMM lesions and p-MLM sign are almost always identified at paracentral location although they often extend outward from the center [30]. In our study, compared with patients without the p-MLM sign, those with the p-MLM sign were significantly older but showed no significant differences in BCVA, sex, or eye distribution (Table 2). It seems that both PAMM and p-MLM sign have similar clinical significance.

Recently, Furashova and Matthè [29] reported that the p-MLM sign could be seen on OCT in 94% of ischemic and 66% of nonischemic RVO cases. In the present study, however, only 24 (seven with RVO and 17 with RAO) of 78 PAMM cases had the p-MLM sign. Evidently, PAMM cases associated with RAO were more likely to present with the p-MLM sign than were those associated with RVO. This may be because the inner synaptic portion of the OPL is more sensitive to retinal arterial ischemia or hypoperfusion than other retinal tissues. All seven eyes with the p-MLM sign that were associated with RVO had skip PAMM lesions. However, in the RAO group, p-MLM sign could be associated with various types of PAMM lesions. This phenomenon was reported previously [11, 12], but the concept of PAMM had not been proposed at the time.

In addition, the hyperreflective line of the p-MLM sign was usually continuous, regardless of the types of PAMM lesions, even with a skip pattern (Figure 1), which is an interesting phenomenon that has not been paid sufficient attention before. According to the concept of misery perfusion [31, 32], reduced oxygenation would preferentially affect the efferent retinal circulation more than afferent retinal circulation, which may cause an ischemic cascade that starts at the level of the deep capillary plexus (DCP) closer to the perivenular pole and manifests as perivenular fern-like PAMM lesions on en face OCT segmentation or skip PAMM lesions on OCT B-scans [16].The inner synaptic portion of the OPL is located at the functional anteroposterior watershed between the DCP and choriocapillaris [33]. Due to the absence of direct blood supply from retinal vessels, oxygen supply of the inner synaptic portion of the OPL mainly originates from oxygen diffusion from the DCP and choriocapillaris. Continuity of the hyperreflective line of the p-MLM sign may be related to vascular components of the DCP. As the hyperreflective line is usually continuous, oxygen saturation in the inner synaptic portion of the OPL may be relatively uniform, which further supports the venous nature of DCP and the serial organization of the three main capillary plexuses in the macula [34]. When milder retinal hypoperfusion occurs, the entire level of the inner synaptic portion of the OPL (near the outer part of the DCP) and INL tissue near the vein develop ischemia and edema, manifesting as a continuous hyperreflective line, p-MLM sign, and skip PAMM lesions. The p-MLM sign may be present in patients with retinal ischemia because of the individual differences in the contribution of choroidal blood supply to the inner synaptic portion of the OPL. Therefore, the role of choroidal blood supply in the pathogenesis of the p-MLM sign should be investigated.

As for the order of occurrence of PAMM and the p-MLM sign, due to rapid progression of RAO, PAMM and p-MLM sign appeared almost simultaneously, or the p-MLM sign was hidden in the hyperreflective inner retina. Relatively, RVO leads to ischemia at a slower rate compared with RAO; therefore, the p-MLM sign may appear first, followed by PAMM. Recently, Browning et al. [35] reported that PAMM could evolve over time into the p-MLM sign. However, the p-MLM sign was evident at the initial visit (Figure 7 in their article) and was rarely found on OCT without other ischemic lesions. This may be because the patient had not undergone the initial examination for a long time, thereby eliciting the impression that the p-MLM sign and PAMM or hyperreflectivity in the inner retina occur simultaneously.

It is a challenge to sufficiently supply the required nutrients to millions of retinal neurons with only a limited blood supply after fulfilling the optical requirement. Evidently, the limited blood supply cannot consistently be received by all retinal neurons. Spatially and temporally heterogeneous capillary perfusion may be the most effective way to deal with this challenge [36]. Such heterogeneous perfusion involves the use of well-controlled intermittent blood redistribution patterns that require strong regulatory capacity and sophisticated structural arrangement of the retinal capillary plexuses. Connections between the retinal capillary plexuses are more complicated than previously thought. However, the results of our study and previous studies demonstrated that the DVC is likely to be subjected to hypoperfusion or ischemia in retinal vascular occlusion presenting with PAMM, the p-MLM sign, or both. These signs alone represent milder ischemia and should prompt clinicians to carry out a comprehensive and meticulous examination to prevent disease progression. If not treated early and effectively, symptoms would further aggravate and even seriously affect the visual prognosis (Figure 2).

This study had several limitations. First, we applied a retrospective study design and did not use a control group. Second, not all patients had undergone FFA; therefore, we could not accurately distinguish between ischemic and nonischemic RVO cases. Finally, follow-up data on ancillary imaging were unavailable for most patients. Nevertheless, this is the largest case series to date describing PAMM and p-MLM sign and the relationship between them in a Chinese clinical setting.

## 5. Conclusions

We found that PAMM occurred at a rate of 5.23% and 19.44% in eyes with RVO and RAO, respectively, in a Chinese clinical setting. In addition, 24 eyes developed both PAMM and p-MLM sign. These signs alone suggest mild ischemia and should prompt us to carry out a comprehensive and meticulous examination to prevent further disease progression and obtain a good prognosis. In addition, the hyperreflective line of the p-MLM sign was usually continuous, which could support the venous nature of the retinal DCP to some extent.

## Figures and Tables

**Table 1:** Patient demographics and ocular findings in PAMM and p-MLM sign.

	RVO	RAO	Total
Eyes (right/left)	29 (21/8)	49 (23/26)	78 (44/34)
Age (range), years	54.00 ± 18.48 (20–78)	64.49 ± 13.90 (23–84)	60.59 ± 16.45 (20–84)
Gender (female/male)	7/22	16/33	23/55
BCVA (range), logMAR	0.86 ± 0.52 (0–2.0)	1.04 ± 0.43 (0.3–2.0)	0.97 ± 0.47 (0–2.0)
Combined with p-MLM sign	7	17	24
Systemic/ocular associations			
Hypertension	17	24	41
Diabetes mellitus	3	7	10
Open-angle glaucoma	6	0	6
No positive medical history	3	18	21

PAMM: paracentral acute middle maculopathy; p-MLM: prominent middle limiting membrane; RVO: retinal vein occlusion; RAO: retinal artery occlusion; BCVA: best-corrected visual acuity; logMAR: logarithm of the minimum angle of resolution.

**Table 2:** Clinical characteristics of PAMM eyes with and without p-MLM sign.

	With p-MLM sign	Without p-MLM sign	*P*
Eyes (right/left)	24 (12/12)	54 (32/22)	0.469^#^
Gender (female/male)	7/17	16/38	1.000^#^
Age (range), years	66.17 ± 11.26 (33–79)	58.11 ± 17.82 (20–84)	0.019^*∗*^
BCVA (range), logMAR	0.98 ± 0.50 (0–2.0)	0.97 ± 0.46 (0.3–2.0)	0.939^*∗*^

p-MLM: prominent middle limiting membrane; BCVA: best-corrected visual acuity; logMAR: logarithm of the minimum angle of resolution. ^#^Fisher's exact test. ^*∗*^Independent sample *t*-test.

**Table 3:** The distribution of three types of PAMM with different etiologies.

	Arteriolar pattern	Globular pattern	Fern-like pattern	Total
BRAO	10	1	2	13
CRAO	12	1	2	15
BRVO	0	1	0	1
CRVO	6^#^	4	9	19
Total	24	7	13	44

^#^Combined with cilioretinal artery occlusion. BRAO: branch retinal artery occlusion; CRAO: central retinal artery occlusion; BRVO: branch retinal vein occlusion; CRVO: central retinal vein occlusion.

## Data Availability

The datasets used and/or analyzed during the current study are available from the corresponding author upon reasonable request.
